# Sensitivity to COVID-19 Vaccine Effectiveness and Safety in Shanghai, China

**DOI:** 10.3390/vaccines9050472

**Published:** 2021-05-07

**Authors:** Jia Lu, Xiaosa Wen, Qi Guo, Mengdi Ji, Felicia Zhang, Abram L. Wagner, Yihan Lu

**Affiliations:** 1 Department of Immunizations, Minhang Centers for Disease Control and Prevention, Shanghai 201101, China; cpulj@126.com (J.L.); xiaosasmmu@163.com (X.W.); qif0713@163.com (Q.G.); 2 Department of Epidemiology, University of Michigan, Ann Arbor, MI 48109, USA; mengdiji@umich.edu (M.J.); fyzhang@umich.edu (F.Z.); 3 Key Laboratory of Public Health Safety (Ministry of Education), Fudan University School of Public Health, Shanghai 200433, China; luyihan@fudan.edu.cn

**Keywords:** COVID-19 vaccination, vaccine hesitancy, China, urban health

## Abstract

Several COVID-19 vaccines have been on the market since early 2021 and may vary in their effectiveness and safety. This study characterizes hesitancy about accepting COVID-19 vaccines among parents in Shanghai, China, and identifies how sensitive they are to changes in vaccine safety and effectiveness profiles. Schools in each township of Minhang District, Shanghai, were sampled, and parents in the WeChat group of each school were asked to participate in this cross-sectional Internet-based survey. Parents responded to questions about hesitancy and were given information about five different COVID-19 vaccine candidates, the effectiveness of which varied between 50 and 95% and which had a risk of fever as a side effect between 5 and 20%. Overall, 3673 parents responded to the survey. Almost 90% would accept a vaccine for themselves (89.7%), for their child (87.5%) or for an elderly parent (88.5%) with the most ideal attributes (95% effectiveness with 5% risk of fever). But with the least ideal attributes (50% effectiveness and a 20% risk of fever) these numbers dropped to 33.5%, 31.3%, and 31.8%, respectively. Vaccine hesitancy, age at first child’s birth, and relative income were all significantly related to sensitivity to vaccine safety and effectiveness. Parents showed a substantial shift in attitudes towards a vaccine based on its safety and effectiveness profile. These findings indicate that COVID-19 vaccine acceptance may be heavily influenced by how effective the vaccine actually is and could be impeded or enhanced based on vaccines already on the market.

## 1. Introduction

The severe acute respiratory syndrome coronavirus 2 (SARS-CoV-2), which causes coronavirus disease [1], has led to substantial morbidity and mortality worldwide and put considerable pressure on public health systems. Since the outbreak, the need for a vaccine, one of the most powerful and cost-effective tools for preventing disease in large populations, has never been more urgent [2]. Unlike previous vaccines, which required years or even decades of clinical trial research, vaccines against COVID-19 were developed at “warp speed” [3,4], much more quickly than previous vaccines [5]. More than 50 COVID-19 vaccine candidates are currently in trials, and several vaccines have already been approved and distributed. By 14 January 2021, more than 35 million doses in 49 countries had been administered [6]. 

A vaccine is only useful if people are willing to receive it. Various countries started rolling out vaccines in late 2020, prioritizing healthcare and other essential workers and those with high-risk health conditions. However, members of the general population may be hesitant to receive a vaccine due to concerns over the speed of its development and concerns about safety and effectiveness. Vaccine hesitancy is already listed as one of the top 10 global health threats, so hesitancy over these new drugs may exacerbate vaccine refusal [7]. According to a survey conducted in 19 countries, 71.5% of the respondents would consider taking a COVID-19 vaccine [8]. This hesitancy and refusal may intensify the pandemic and put more pressure on health systems.

A previous study showed that respondents from China showed the highest positive response (88.6%) and lowest negative response (0.7%) when asked if they would accept a “proven, safe and effective COVID-19 vaccine” [8]. However, sensitivity to different levels of effectiveness and safety within China was still unclear. It is also not clear what factors may have influenced their acceptance. It is crucial to consider the public’s acceptance of vaccines that have different levels of safety and effectiveness and the factors related to them to adopt evidence-based interventions for varying vaccine levels to counter future outbreaks. This study characterizes vaccine hesitancy towards the COVID-19 vaccine among parents in Shanghai, China, and identifies how sensitive they are to changes in the vaccine safety and effectiveness profile. 

## 2. Materials and Methods

### 2.1. Study Population

In this study, a stratified cluster sampling method was used to conduct a questionnaire survey in each of the 13 townships in Minhang District, Shanghai. We wanted a sample size of 2345 to have a margin of error of at least 2% for our outcome: the proportion who would accept a given vaccine with alpha of 0.05. We obtained a larger sample given the ease at obtaining data within schools. Within each township, a convenience sample of one school was chosen. In each sampled school, a health instructor sent the questionnaire link to each class’s WeChat group. Following this, the parents of the students filled out the questionnaire. In order to improve the controllability of the questionnaire’s source, the fidelity of the sampling, and the participation of the parents, researchers answered any parents’ questions live during the survey completion; questionnaires that were completed in less than 5 min (estimated time) were excluded from the data analysis. The questionnaire was developed by staff at the Minhang Centers for Disease Control and Prevention.

### 2.2. Derived Variables

**Figure 1:**
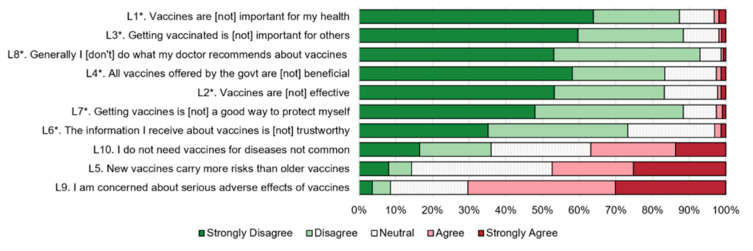
Responses to questions about vaccine hesitancy among parents of school-aged children in Shanghai, China, 2020. Questions with an asterisk (*) have been reverse coded so that all questions have responses with higher values being more vaccine hesitant.

Vaccine hesitancy was assessed through a 10-item adult Vaccine Hesitancy Scale (aVHS) (Figure 1). This questionnaire had been validated by U.S. and Chinese samples [9], and within this sample there was high internal reliability of this scale (Standardized Crohnbach’s alpha = 0.82). Briefly, each item was based on a 5-point Likert scale: a score of 1 represented the lowest degree of vaccine hesitancy and 5 the highest). The results were summed for a possible range of 10 to 50. A score of 10 to 24 was categorized as “not hesitant”, while 25 to 50 was considered “hesitant” [9].

We assessed acceptance of a vaccine first by providing participants with different profiles of effectiveness (95, 60 and 50%) and safety (5, 10, and 20% risk of fever). We then asked parents if they would accept a vaccine with a given profile for themselves, for their child, or for an elderly parent. From this information, we also if someone were sensitive to vaccine effectiveness (i.e., they would accept 95% but not 50% effectiveness) or safety (i.e., they would accept a 5% risk but not a 20% risk of fever). Across each characteristic (effectiveness and safety) individuals fell into one of three categories: they would not accept a vaccine under any circumstance; they were sensitive to the profile; or they would accept any vaccine.

Demographic characteristics of the parents, including their age, second child, and stated relative income in their peer group, were also collected.

### 2.3. Statistical Analysis

After quantifying the proportion of individuals with sensitivity to vaccine effectiveness and safety, we created two multivariable models in which the outcomes were the three-level characteristics of sensitivity to effectiveness and safety. The primary independent variable was vaccine hesitancy, as measured by the aVHS. We also included mother vs father, age of parent, presence of second child, age of first child, sex of first child, and stated relative income as confounders in this analysis based on an a priori consideration of these variables with vaccine hesitancy and profile sensitivity. This model output odds ratios (ORs) and 95% confidence intervals (CIs). Data were analyzed in SAS version 9.5 (SAS Institute, Cary, NC, USA).

## 3. Results

Overall, 3673 parents responded to the survey. Demographic characteristics of the parents are shown in Table 1. Most respondents (69.1%) were mothers. A plurality (37.1%) had their first child at 25 to 29 years of age; most (67.2%) did not have a second child; and for less than half (45.3%), the first child was elementary aged (6–11 years old).

Responses to vaccine hesitancy items are shown in Figure 1. Individuals expressed a great deal of concern about serious adverse effects (40.1% agreed and 30.1% strongly agreed), believing that newer vaccines carried more risks than older vaccines (22.2% agreed and 25.2% strongly agreed), and that vaccines for diseases that were no longer common were not needed (23.1% agreed and 13.8% strongly agreed).

Overall, 29.5% (1083) were vaccine hesitant, as measured by the aVHS, with some trends by demographic group. Individuals who were older when they had their first child (32.1% of those aged 30–45 years) were more hesitant compared to of those who were 18–22 (19.4%) when they had their first child, (*p* < 0.0001). Additionally, those with a second child were less vaccine hesitant (24.9%), compared to those with only one child (31.4%), (*p* < 0.0001).

Acceptance of a COVID-19 vaccine varied according to its safety and effectiveness profile: the highest levels of acceptance were for 95% effectiveness and a 5% risk of fever; and the lowest were for 50% effectiveness and a 20% risk of fever (Table 2). Almost 90% would accept a vaccine for themselves (89.7%), for their child (87.5%) or for an elderly parent (88.5%) if it had the highest attributes. For vaccines with the lowest attributes, these numbers dropped to 33.5%, 31.3%, and 31.8%, respectively.

Overall, about 10% of individuals would not accept a vaccine, regardless of its safety or effectiveness profile; almost half (48.6%) were not sensitive to vaccine effectiveness; and 31.3% would accept a 95% effective vaccine, but not a 50% effective one. There was less sensitivity to vaccine safety as measured by risk of fever. Almost two-thirds, 63.3%, would accept a vaccine regardless of the risk of fever, and about one-fourth, 26.8%, would accept a vaccine with a 5% risk of fever but not a 20% risk.

Sensitivity to vaccine safety and effectiveness was significantly related to vaccine hesitancy, age at first child’s birth, and stated relative income (Table 3). Having a second child was significantly related to sensitivity to vaccine effectiveness (*p* = 0.0334), but not safety (*p* = 0.0998). For example, those who were vaccine hesitant were 10.47 times more likely not to accept a vaccine, and 2.60 times more likely to be sensitive to vaccine effectiveness, compared to those who were not vaccine hesitant (*p* < 0.0001). Vaccine hesitancy was associated with greater odds of not accepting any vaccine or being sensitive to the risk of fever (*p* < 0.0001). Those who were younger at their first child’s birth had reduced odds of not accepting a vaccine or being sensitive to its effectiveness or safety profile (*p* < 0.0001 for effectiveness, *p* = 0.0055 for safety). Those whose income was less than average were less sensitive, both to the effectiveness profile (*p* = 0.0035) and to the safety profile (*p* = 0.0067).

## 4. Discussion

Safety and effectiveness are the two most important indicators for evaluating a new vaccine, and new vaccines undergo substantial tests of their safety and effectiveness before and after coming onto the market [10,11]. Previous studies showed that most parents express concerns about side-effects, safety, and effectiveness [12]. These concerns may be even more prominent for the COVID-19 vaccine, based on the perceived speed of vaccine development. Similarly, parents showed a substantial shift in attitudes towards the COVID-19 vaccine based on safety and effectiveness. The majority of the respondents would accept a vaccine with high levels of safety and effectiveness, but only one-third would accept a vaccine with lower levels of safety and effectiveness. These preferences could hamper acceptance of the vaccine. Interestingly, the public showed a different level of sensitivity toward safety and effectiveness, with more sensitivity towards effectiveness.

Although vaccines are currently available in some locations, safety and effectiveness may vary. For example, for two that were approved in the U.S., Pfizer-BioNTech’s vaccine was 95% and Moderna’s 94.1% efficacious in preventing the COVID-19 disease [13,14]. The AstraZeneca vaccine used in the U.K., India, and Mexico was reported to have an average efficacy of 70% [15]. For the inactivated vaccines produced by Chinese pharmaceutical companies, efficacy ranged from 50 to over 90%, depending on the considered outcome and study site [16]. Currently, 89 vaccines are being tested in clinical trials, and 27 have reached the final stage [17]. As more vaccines come onto the market, the public may find itself choosing among vaccines that have widely varying levels of efficacy.

The study found a strong relationship between vaccine hesitancy and COVID-19 vaccination, and the respondents believe that the new vaccine carried more risks than the older vaccine. The role of vaccine hesitancy, and anti-vaccine movements, has been previously explored. For instance, Gaulano et al. found that Italian women who received information from anti-vaccination movements were less likely to accept mandatory vaccines [18]. However, it is essential to note that people might be hesitant about the COVID-19 vaccine but not for vaccines in general. The COVID-19 vaccine went through the process from development to distribution worldwide at “warp speed.” It also adopted a new approach of using mRNA, which is different from traditional vaccines that use weakened or inactive components of the pathogen [19]. Scientists and governments are still assessing the effectiveness after the COVID-19 vaccine was authorized for emergency use in the U.S. [20]. 

COVID-19 vaccine hesitancy is present not only in the general public, but also among healthcare workers. A recent survey by Kaiser Family Foundation found that nearly a third of the healthcare workers would probably or definitely refuse the vaccine [21]. Healthcare workers expressed concerns about not enough research having been done; the lack of adequate transparency among pharmaceutical companies, research companies, and governments; and fear of being part of another “Tuskegee Study” [22]. Thus, how to break through the vaccine hesitancy among healthcare workers, who have a higher risk of contracting the virus and play important roles in their patients’ vaccine decision making, is of the utmost importance. Even in non-pandemic settings, health care workers have relatively low coverage of non-mandatory vaccines, and this varied by age, with younger personnel more likely to be vaccinated [23].

### Strengths and Limitations

This is a cross-sectional study and so we were unable to look at longitudinal connections. Additionally, we assessed intent to get a vaccine, but actual vaccine acceptance may differ as more information becomes available. The vaccine effectiveness and safety profiles that we chose were based on possible ranges from existing influenza and measles vaccines, but the actual characteristics of COVID-19 vaccines may differ. We also did not evaluate acceptance or hesitancy towards other vaccines routinely provided to children, such as the mumps–measles–rubella vaccine, and did not adjust our analyses for this variable. Nonetheless, using a large sample of parents, we were able to assess variations in vaccination intent using a validated vaccination hesitancy scale.

## 5. Conclusions

In this study of parents of school-aged children in a suburb of Shanghai, parents showed a substantial shift in attitudes towards the COVID-19 vaccine based on the vaccine’s safety and effectiveness. The majority of respondents would accept a vaccine with the most ideal levels of safety and effectiveness, but only one-third would accept vaccines with the least ideal attributes. These findings indicate that COVID-19 vaccine acceptance may be substantially influenced by how effective the vaccine actually is. Controlling outbreaks of COVID-19 in the presence of these strong preferences would require substantial use of non-pharmaceutical interventions.

Local circumstances are important to consider when developing programs to promote vaccines, as thoughts about different aspects of vaccination are not uniform across countries. We did not find consistent associations about education and vaccine hesitancy, in contrast to prevailing findings about this relationship in high income countries; more work needs to be done on fully understanding socio-cultural influences on vaccine decision-making. Continued surveillance of attitudes towards vaccination in low- and middle-income countries can help identify shifts in future opinions in vaccination attitudes.

## Figures and Tables

**Table 1:** Demographic characteristics of a sample of Shanghai parents of school-aged children, 2020.

Characteristic	Category	Count (Column %)	Vaccine Hesitant (Row %)	*p*-Value
Relation to child	Mother	2538 (69.1%)	762 (30.0%)	0.3162
	Father	1093 (29.8%)	306 (28.0%)	
	Other	42 (1.1%)	15 (35.7%)	
Age at first child’s birth	18–22 years	346 (9.9%)	67 (19.4%)	<0.0001
	23–25 years	837 (24.0%)	209 (25.0%)	
	26–29 years	1291 (37.1%)	418 (32.4%)	
	30–45 years	1010 (29.0%)	324 (32.1%)	
Second child	No	2412 (67.2%)	758 (31.4%)	<0.0001
	Yes	1177 (32.8%)	293 (24.9%)	
Age of first child	0–5 years	35 (1.0%)	9 (25.7%)	0.0702
	6–11 years	1624 (45.3%)	468 (28.8%)	
	12–14 years	1133 (31.6%)	338 (29.8%)	
	15–17 years	555 (15.5%)	181 (32.6%)	
	≥18 years			
Sex of first child	Male	1844 (50.8%)	537 (29.1%)	0.6950
	Female	1787 (49.2%)	531 (29.7%)	
Stated relative income	Less than average	424 (11.5%)	146 (34.4%)	0.0588
	About average	2710 (73.8%)	783 (28.9%)	
	More than average	539 (14.7%)	154 (28.6%)	

**Table 2:** Acceptance of a COVID-19 vaccine, based on the safety and effectiveness profile.

Sensitivity	Condition	For Self	For Child	For Parent
Acceptance of a vaccine based on effectiveness and safety profile	95% effective, 5% risk of fever	3294 (89.7%)	3213 (87.5%)	3250 (88.5%)
	95% effective, 20% risk of fever	2330 (63.4%)	2164 (58.9%)	2154 (58.6%)
	60% effective, 10% risk of fever	1662 (45.3%)	1569 (42.7%)	1567 (42.7%)
	50% effective, 5% risk of fever	1790 (48.7%)	1708 (46.5%)	1716 (46.7%)
	50% effective, 20% risk of fever	1230 (33.5%)	1151 (31.3%)	1166 (31.8%)
Sensitivity to COVID-19 vaccine effectiveness	Would not accept any vaccine	369 (10.1%)	453 (12.4%)	413 (11.3%)
	Would accept 95% effective vaccine, not 50%	1514 (41.3%)	1512 (41.2%)	1544 (42.2%)
	Would accept any vaccine	1780 (48.6%)	1701 (46.4%)	1706 (46.6%)
Sensitivity to COVID-19 vaccine safety	Would not accept any vaccine	363 (9.9%)	445 (12.2%)	405 (11.1%)
	Would accept vaccine with 5% risk of fever, not 20% risk	980 (26.8%)	1064 (29.1%)	1114 (30.5%)
	Would accept any vaccine	2314 (63.3%)	2149 (58.8%)	2136 (58.4%)

**Table 3:** Sensitivity to COVID-19 vaccine effectiveness and safety in multinomial logistic regression models among Shanghai parents of school-aged children, 2020.

Characteristic	Compared to Those Who Would Accept a Vaccine, Regardless of Effectiveness		Compared to Those Who Would Accept a Vaccine, Regardless of Risk of Fever	
	Would Not Accept Any Vaccine, OR (95% CI)	Would Only Accept 95% Effective Vaccine, OR (95% CI)	Would Not Accept Any Vaccine, OR (95% CI)	Would Only Accept Vaccine With 5% Risk of Fever, OR (95% CI)
Vaccine hesitant				
No	ref	ref	ref	ref
Yes	10.47 (8.03, 13.67)	2.60 (2.19, 3.09)	8.45 (6.54, 10.91)	2.48 (2.09, 2.94)
Relation to child				
Mother	ref	ref	ref	ref
Father	1.14 (0.87, 1.51)	0.93 (0.79, 1.10)	1.09 (0.83, 1.42)	0.84 (0.70, 1.00)
Age at first child’s birth				
18–22 years	0.46 (0.27, 0.79)	0.43 (0.32, 0.58)	0.55 (0.32, 0.94)	0.53 (0.38, 0.74)
23–25 years	0.52 (0.36, 0.75)	0.54 (0.43, 0.66)	0.67 (0.47, 0.97)	0.79 (0.63, 0.99)
26–29 years	0.78 (0.57, 1.05)	0.82 (0.68, 0.99)	0.81 (0.60, 1.09)	0.85 (0.70, 1.03)
30–45 years	ref	ref	ref	ref
Have a second child				
No	ref	ref	ref	ref
Yes	0.75 (0.55, 1.01)	0.83 (0.70, 0.98)	0.81 (0.61, 1.09)	0.84 (0.70, 1.01)
Age of first child				
0–5 years	1.37 (0.45, 4.18)	0.67 (0.31, 1.43)	1.65 (0.56, 4.85)	0.82 (0.36, 1.84)
6–11 years	ref	ref	ref	ref
12–14 years	1.16 (0.86, 1.55)	1.03 (0.87, 1.22)	1.14 (0.85, 1.51)	0.97 (0.81, 1.16)
15–17 years	1.26 (0.88, 1.79)	0.92 (0.74, 1.15)	1.26 (0.89, 1.79)	0.84 (0.66, 1.06)
≥18 years	1.44 (0.80, 2.60)	0.89 (0.63, 1.26)	1.33 (0.74, 2.39)	0.76 (0.51, 1.14)
Gender of first child				
Male	ref	ref	ref	ref
Female	0.94 (0.73, 1.21)	1.09 (0.94, 1.26)	0.87 (0.68, 1.12)	0.97 (0.83, 1.13)
Stated relative income				
Less than average	0.86 (0.59, 1.28)	0.66 (0.52, 0.84)	1.00 (0.69, 1.46)	0.69 (0.53, 0.91)
About average	ref	ref	ref	ref
More than average	1.39 (0.99, 1.96)	1.05 (0.85, 1.29)	1.50 (1.08, 2.09)	1.14 (0.92, 1.42)

## Data Availability

The data presented in this study are available on request from the corresponding author. The data are not publicly available due to personal information contained in some of the data fields.
